# Migration and mental health in Europe (the state of the mental health in Europe working group: appendix 1)

**DOI:** 10.1186/1745-0179-1-13

**Published:** 2005-08-31

**Authors:** Mauro Giovanni Carta, Mariola Bernal, Maria Carolina Hardoy, Josep Maria Haro-Abad

**Affiliations:** 1 Department of Public Health University of Cagliari, Italy; 2 Unitat de Recerca i Desenvolupament, Hospital Sant Joan de Deu-SSM, Barcelona, Spain

## Abstract

**Background:**

This paper is a part of the work of the group that carried out the report "The state of the mental health in Europe" (European Commission, DG Health and Consumer Protection, 2004) and deals with the mental health issues related to the migration in Europe.

**Methods:**

The paper tries to describe the social, demographical and political context of the emigration in Europe and tries to indicate the needs and (mental) health problems of immigrants. A review of the literature concerning mental health risk in immigrant is also carried out. The work also faces the problem of the health policy toward immigrants and the access to health care services in Europe.

**Results:**

Migration during the 1990s has been high and characterised by new migrations. Some countries in Europe, that have been traditionally exporters of migrants have shifted to become importers. Migration has been a key force in the demographic changes of the European population. The policy of closed borders do not stop migration, but rather seems to set up a new underclass of so-called "illegals" who are suppressed and highly exploited. In 2000 there were also 392.200 asylum applications.

The reviewed literature among mental health risk in some immigrant groups in Europe concerns: 1) highest rate of schizophrenia; suicide; alcohol and drug abuse; access of psychiatric facilities; risk of anxiety and depression; mental health of EU immigrants once they returned to their country; early EU immigrants in today disadvantaged countries; refugees and mental health

Due to the different condition of migration concerning variables as: motivation to migrations (e.g. settler, refugees, gastarbeiters); distance for the host culture; ability to develop mediating structures; legal residential status it is impossible to consider "migrants" as a homogeneous group concerning the risk for mental illness. In this sense, psychosocial studies should be undertaken to identify those factors which may under given conditions, imply an increased risk of psychiatric disorders and influence seeking for psychiatric care.

**Comments and Remarks:**

Despite in the migrants some vulnerable groups were identified with respect to health problems, in many European countries there are migrants who fall outside the existing health and social services, something which is particularly true for asylum seekers and undocumented immigrants. In order to address these deficiencies, it is necessary to provide with an adequate financing and a continuity of the grants for research into the multicultural health demand. Finally, there is to highlight the importance of adopting an integrated approach to mental health care that moves away from psychiatric care only.

## 1. Migration in Europe: the social, demographic and political context

### Trends in migration in Europe

The number of migrants in the world has more than double since 1975, with most living in Europe (56 million), Asia (50 million) and Northern America (41 million) [1]. In 1990, migrants accounted for over 15% of the population in 52 countries. Most of the migration was from developing to developed countries, even though migration within developing countries is also increasing as the pace of economic growth and the demand of labour between these countries also changes [2].

In Europe, mass migration is not new. During the 20 th century Europe has experienced three major periods of movements: around the time of the First and Second World Wars and during last decade. Migration during the 1990s has been high. It is a period characterised by new migrations, especially from the Eastern and Central European countries and from the Commonwealth of Independent States, but it is the Balkans war, which has dominated these movements [3]. Some countries in Europe, like Ireland, Spain, Italy or Portugal, that have been traditionally exporters of migrants have shifted to become importers. Given the growing economic and political cooperation emerging between many European countries, competition for highly-skilled labour will intensify and the demand of more labour movement will surely continue to grow as organisms such as the International Organisation for Migration predicts.

The role of migration in European population change is being debated during recent years as a result of growing concerns about issues such as demographic ageing, shortages of working age populations and payment of pensions among others [4]. The United Nations Population Division has suggested that Europe might need replacement migration to cope with this potential problems, ranging from around one to 13 million new immigrants per year from 2000 to 2050 [5].

Migration has been a key force in the demographic changes of the European population during last decade. Specifically, the components of population change for the period 1997–99, indicate that migration was the most important component in 33 per cent (15 out of 46) of the countries. 33 countries experienced an increase in population during that period, while 13 had a decrease. Of the 33 countries which had an increase in population, 25 had a net gain of population through migration [3]. The total recorded stock of foreign population living in Europe in 1999/2000 was 21.16 million people, constituting a 2.6% of the aggregate population in Europe [3]. This figure might surely have increased during recent years as immigration rates in many countries have not ceased to grow.

Nevertheless, within Europe there exist great differences in the pattern of migration depending on the countries. This way, some experts have come to distinguish a Northern and a Southern model of immigration in Europe. The Northern model would be composed of those Northern European countries (e.g. United Kingdom, Netherlands, Germany and Sweden among others), which have had a long experience of immigration through history, and especially right after Second World War, when arrival of immigrants experienced a sharp increase. The Southern model would comprise Southern European countries for which the immigration phenomenon is relatively recent, such as Spain or Portugal. While in Spain the percentage of foreign 'legal' residents sum up to 3.2% of total population in 2002, in other European countries this percentage fluctuates between 5–10% at this time [5].

The composition of immigration by countries is also very different. The vast majority of immigrants in Central and Eastern Europe and Scandinavian countries come from elsewhere in Europe. Germany's immigration field is also strongly European, and along with Austria and Finland receives a high proportion of its immigrants from Central and Eastern Europe. In contrast, the Mediterranean countries, together with UK and Netherlands, attract a high proportion of immigrants beyond Europe. Almost a third of UK's and Spain's immigrants come from outside Europe [3].

Political and socio-economic instability in and around Europe has significantly increased the number of refugees and asylum seekers arriving in European countries.

A refugee is "one who flees to a foreign country or power to escape danger or persecution" [6]. Although the UNHCR statistics are based on this definition, many agencies reserve the term 'refugee' for those whose application for asylum under the terms of the Refugee Convention has been accepted. This is to distinguish them from 'asylum seekers', who still have to prove their right to asylum, and 'illegal aliens', who may be fleeing from danger or persecution, but have not entered the official asylum procedure or have been rejected by it.

According to United Nation High Commission for Refugees (UNHCR) in 2000 there were 392.200 asylum applications in Europe. Northern European countries have been receiving for a longer period of time a great population of refugees, while countries from Southern Europe receive a vast majority of economic migration. However, the low numbers of asylum seekers in Southern European countries may be misleading owing to the large amount of refugees that are thought to by-pass the step of applying for asylum, then getting the residence permit through immigration laws or becoming "illegal aliens" [7].

The presence of undocumented immigrants is a well-established fact in most European countries. They come or are "called" into Europe to perform badly paid, physically and psychologically stressful jobs in highly qualified service economies and welfare states. The closure of frontiers to new immigration has not prevented the increase of "illegal aliens" in Europe. On the contrary, the welfare gap between Europe and its neighbouring countries makes such jobs more attractive for enterprising women and men in the poorer and poorest regions of the world. According to the International Labour Office estimates, in 1991 there were around 2.6 million of undocumented immigrants living in Europe [3]. This is the last official data estimated. Other sources nowadays estimate the number of undocumented immigrants in Europe in more than 3 million [8], finding the largest populations in the Southern European countries due to their structure, culture, history and geography [5]. One of the features of this structure, present in the South of Europe, is the great expansion of the *black economy*. Although there is no way to confirm these numbers, the trend seems to go upward.

### Policy and political context of migration in Europe

Trends in migration in Europe commenced to transform a few decades ago as the result of changes that were taken place in the economic, political and social realms. This inflection point was described by Massey et al [9] to take place during the 1970s, when patterns of migration meant a rupture to those of all past migration movements. Among the new features of these new migrations we find, on behalf of the immigrants, the fact that they are not simply looking for an immediate job but also for situations which give them a higher quality of life, together with better future possibilities for themselves and their children; on behalf of the reception countries, the development of a labour legislation before unknown which have given place to a clear contradiction between legal and illegal work [10]. These trends, which have changed the profile and perception of international migration that marked the post war era, have become especially visible during the 1990s. Due to this new model of migration, characterised for the change into a post-industrial society, there is an urgent need for the elaboration of policies that respond to this new global situation.

EU member states have been practicing a policy of closing borders throughout the 1990s, a policy that have been specially hardened during recent years. Different strategies for restricting the entrance of immigrants have been developed by the different countries. For example, in Belgium, Italy and Spain, the states have mixed restrictive immigration (e.g. establishing a quota system for work-related migration) policies with recurrent processes of regularisation of undocumented immigrants, while in Germany, the state has opted for restricting immigration via the mechanism of political asylum, ignoring in this way the increasing number of undocumented immigrants http://www.freudenbergstiftung.de.

However, the policy of closed borders do not stop migration, but rather seems to set up a new underclass of so-called "illegals" who are – against all declarations of human rights – inhumanely suppressed and highly exploited. Inconsistency and the lack of a future vision have marked the immigration and asylum policies of most European countries for the last fifteen years.

The EU is still in its way to elaborate a common policy and therefore, determine its immigration model. For that the 7^th ^Conference of Minister responsible for Migration Affairs was held in Helsinki during September 2002. Nevertheless, some sources point to the possible election of a *model of (work-related) quotas*, a model that has been widely criticized because of its proved failure up to now. An active and common policy of immigration not only focused on control but also on integration policies, is needed in Europe, since immigration has never stopped and will continue.

## 2. Needs and (mental) health problems of immigrants

Among all the changes a human being must face throughout his live, few are so wide and complex as those which take place during migration. Practically everything that surrounds the person who emigrates changes. Aspects ranging from diet, family and social relations to climate, language, culture, and status are subject to change. The decision to migrate originates in a perceived lack of prospects that a person has in his own country. Every person who emigrates experiences affective loss, but is buoyed up in the hope of finding the *first world paradise *they often know so little about.

The singularity of the migratory experience lies in the fact that it is a psycho-social process of loss and change, which is known in the psychiatry of migration as a grief process [11]. The grief is considered as a type of stress characterised by its intensity and length [12]. The process of migration has been explained through a model comprised of seven griefs (losses) causing anguish that a person will experience with time: family and friends, language, culture, homeland, loss of status, loss of contact with the ethnic group, and exposure to physical risks. Furthermore, the migratory grief process is partial, as the grief's subject do not really disappear (unlike the grief for the death of a loved one), and recurrent, as the contact with the country of origin could revive the bonds [13]. Difficulties in expressing grief can cause psychological problems. These difficulties are accentuated when migration is accomplished under adverse conditions. The reception in the new country is crucial for the complete and successful elaboration of the grief process.

In the case of refugees, who have to flee their country for fear of being persecuted, the grief process is more complex. There is the fact that they can not go back to their country of origin, so that their grief is, in this case, closer to the experience of loss, than to that of separation. War-related experiences and occupational status before migration have been proved to be risks factors for psychiatric symptoms among Somali refugees living in the UK [14]. As for every immigrant, the post-migration environment has been proved to have a considerable influence regardless of prior traumatic exposures, being the level of affective social support in exile an important determinant of the severity of possible disorders [15].

As a diagnosed condition Post Traumatic Stress Disorder (PTSD) is by far the most common mental health problem among refugees and asylum seekers [16], followed by mood disorders. According to the Medical Foundation in the UK, the range of psychological problems experienced by torture survivors can include: nightmares, hallucinations, panic attacks, sexual problems, phobias, difficulty in trusting others and forming relationships and depressive illness or anxiety [16].

A study carried out in Portugal with asylum seekers and refugees found that principal sources of stress of this population were connected to traumatic or harrowing experiences in the country of origin due to political or family reasons, problems of communication (language), legal status and work.

In Spain, researches [17-19] have been carried out on mental and psychosomatic disorders in immigrant populations. These researches found the following factors associated with the immigrants' mental health: labour and economic instability, cultural and social marginalisation, family estrangement, pressures to send money to their families, racial discrimination and lack of statutory documentation [20].

In spite of these considerations, which seem to evidence that it is impossible to consider "migrants" as a homogeneous group concerning the risk for mental illness according to Murphy's hypothesis [21], many studies seem to confirm that not a generalized psychopathological risk is present among emigrants. In this sense, psychosocial studies should be undertaken to identify those factors which may, under given conditions, imply an increased risk of psychiatric disorders and influence seeking for psychiatric care [22]. Specific migrant situations may be evaluated, concerning psychopathological risk and seeking for psychiatric care, in order to variables as: motivation to migrations (see Rack work subdividing settler, refugees, gastarbeiters, [23]; distance for the host culture (religion, language, and so); ability to develop mediating structures [24]; legal residential status.

### The Chronic and Multiple Stress Syndrome (Ulysses syndrome)

The particularly hard conditions of today's migration seem to be propitiating a worsen in the mental health of the newcomers. Current situations are making of the migratory experience, an extremely hard and unbearable process. Thousands of people are emigrating and suffering levels of stress, in some cases, inhuman. Examples of this, it's the situation given in the South of Europe particularly Spain and Italy, where in recent years, the coasts of Sardinia, Sicily, Southern Spain and the Canary Archipelago have registered a massive arrival of small, fragile crafts which hundreds of Africans use to get into European territory putting their lives in danger [20].

Psychiatrists from the Psycho-pathological and Psycho-social Assistance Service (SAPPIR) team, located in Barcelona, have described the common symptoms that most immigrants present when attending the centre and have called it *Chronic and Multiple Stress Syndrome *in immigrants (or *Ulysses syndrome*), relating the risky and hard journey that the immigrants pursue in search of a better life with the odyssey of the mythical Greek character in his long voyage through the Mediterranean. Immigrants affected by this syndrome present depressive symptomatology with atypical characteristics, where depressive symptoms are mixed with anxious, somatoform and dissociative symptoms [13]. The development of this condition occurs progressively as the immigrant encounters the obstacles that take place during the migration process: dangerous journey, distance from their own environment and family, difficulties to find a job, food, housing and obtain documents, and the racism suffered in the reception country. The psychiatrist's team propose that the '*Chronic and Multiple Stress Syndrome*' should constitute an autonomous category situated in between adjustment disorders and Posttraumatic stress disorder [13].

The growing incidence of this syndrome in many psychiatrist and psychological services across Europe have alerted a group of social scientists and health care professionals from different countries, who have addressed to the EU Parliament, in order to make an urgent call about the situation which it has been worsening during last five years [8].

## 3. Epidemiological studies

The first epidemiological evaluations of psychiatric disorders in immigrants date back to more than seventy years ago [25]. However, the results obtained in these studies were somewhat limited due to a number of methodological problems which have only recently been overcome thanks to the introduction of standardized diagnostic criteria and structured interviews and to investigations being carried out on the general population and not only on subjects who had been seen by health professionals [26].

Even so, currently in Europe epidemiological studies which offer information on mental health status of immigrants are still very scant. Most studies carried out until nowadays are clinical and mostly limited to private medical practices, as epidemiological research offers by now little information. These clinical studies have some limitations due to the use of small and, frequently, biased samples.

There is little data at our disposal with regard to the level of psychic and physical health among those who are culturally different owing to inadequate systems of registration (e.g. lack of the 'nationality' variable). These systems have recently started to be modified in some countries in order to provide soon with useful data on the health profile of the newcomers.

Nevertheless, some epidemiological studies do exist although its distribution across UE countries is very patchy. For example, while in some countries such as the United Kingdom, Netherlands and Sweden there are some data concerning the mental health status of immigrants (including in this group asylum seekers and refugees), in Belgium there are no nationwide or regional epidemiological studies available on immigrants, as neither, for example, in Spain or Portugal.

The following review – clustered by countries – underline the different trend of research at present time in evolution in each European country. Some topics as drug consumption in immigrated people and psychiatric health in refugees were summarized in a transnational way. Some of these studies were highlighted in the Council of Europe's report on *Health Conditions of migrants and refugees in Europe *[27]. The more relevant research concerning Refugees from Balkans was from USA (Harvard programme from refugees), it was summarized considering the relevance of this health problem for the European Union.

### - United Kingdom

• Hospital admission rates for schizophrenia are highest for people with of Caribbean, Irish and Polish background [28-32] with special high incidence in 16–29 year old people of Caribbean background. Also higher rates than the indigenous are found in people from Indian and Pakistan. Two studies collecting rates of first onset schizophrenia were recently carried out in Trinidad and London [33], the results confirm that sending countries have low rates of schizophrenia. Author indicates that the impact of migration itself produces high stress but rates of schizophrenia are even higher in the second generation, suggesting that other social factors may be responsible for the increase. Social factors and genetic vulnerability may play a role considering that only sub groups of migrants show higher rates of psychosis. A review of the literature about seventeen population based studies [34] proposed that the developmental task for formulating the life plan challenges the young adult's executive function abilities, which may be weaker in individuals vulnerable to schizophrenia. Formulating the life plan may be made more difficult by the position of disadvantaged ethnic minorities, raising the risk for schizophrenia. A more recent review [35] indicates that African-Caribbean population in England is at increased risk of both schizophrenia and mania and the excess of the two psychotic disorders are probably linked: African-Caribbean patients with schizophrenia show more affective symptoms, and more relapsing course with greater social disruption but fewer chronic negative symptoms, than White patients.

• Suicide rates of young women immigrants from the Indian subcontinent are consistently higher than those of their male counterparts and of young women in the indigenous populations of the countries to which they immigrated [36,37]. Family conflict appears to be a precipitating factor in many suicides, whereas mental illness is rarely cited as a cause. Depression, Anxiety and domestic violence may contribute to the high rates. Authors suggest that affective disorders may be underdiagnosed in this population.

• The issue concerning the lower rate of recognized mental disorders in women of Indian origin was addressed by a survey of Jacob and coll. carried out in a general practice setting in West London [38]. Common mental disorders were documented in 30% of patients (similar in Indian women to those in other UK populations), individuals with common mental disorders had a higher frequency of consultation, were less likely to see depression as an indicator for medical intervention and were more likely to withhold some of their concerns from the general practitioner (GP). Incorrect diagnosis by the GP was most likely to occur when patients did not disclose all their complaints. Authors conclude that differing conceptualisation of common mental disorders may contribute to their under-recognition in women of Indian origin. A recent survey examined the contribution of Asian ethnicity in rates of practice prescribing for antidepressant and anxiolytics medication in 164 general practices in East London [39]. Results indicate that where the proportion of Asian patients is high, both antidepressants and anxiolytics prescribing is low. The study confirms that the putative low rates of non-psychotic disorders attribute to Asian population is not a selective feature of access to secondary care but uncertainty remains as the low rate of antidepressants and anxiolytics prescription of GP may be due to a lower prevalence rate, or to "culture-bound syndromes" or to practical difficulties in diagnosis and management within the general practice setting.

• The alcohol abuse among people of Indian descent is reflected in rates of cirrhosis-related mortality, which are twice as high as among English males [40]. Drug abuse has also been reported as a problem [41].

### - The Netherlands

• According to UK studies a series of researches conducted in the Netherlands indicate that the incidence [42] and point prevalence [43] of schizophrenia is increased in several (Morocco, Suriname and Netherlands Antilles) but not all (Turkish and Western countries) immigrants groups. The relative risk of schizophrenia in Suriname born immigrants against Suriname resident born population was 1.46 [42]. Authors conclusion is that selective migration according to Odegaard's selection hypothesis cannot solely explain the higher incidence of schizophrenia in Surinamese immigrants to the Netherlands.

• Drug abuse related schizophrenia together with psychotic diseases was found not uncommon among some migrant groups [44], especially migrants from Suriname and the Antilles. It has been suggested as an explanation for the increased incidence of psychosis among some immigrant groups. A recent population-based, first contact incidence study found misused of illicit substance in: 23% of Dutch, 17% of Moroccans, 27% of Surinames and 30% of Turkish. These results seem to suggest that a higher rate of drug misuse is an unlikely explanation for the increased incidence of psychotic disorders among Moroccan and Surinamese immigrants in the Netherlands [45].

• In a country where the unemployment rate among migrants was almost three times that of the nationals in 1994, the suicide rate among children of immigrants was considerably higher than that of the national population [46].

• A recent study carried out in randomly selected sample of Turkish immigrants, found a prevalence rate of "minor" psychiatric disorders (33.4%, 36.1% in females, 27.9% in males), higher than those normally found in community based samples. The results suggest that the expression of somatic complaints around "tightness" should alert physicians to further explore symptoms of minor psychiatric disorders and to examine sources of distress stemming from the partner relationship, the family, the work and from the poor housing and financial conditions [47].

• A study on service utilisation in women immigrants in Amsterdam found that Surinamese, Antillean, Turkish and Moroccan women made considerably less use of mental health care services than native born women. Immigrant women consulted, on the contrary, social work facilities and women crisis intervention centres nearly 1.5 times more than mental health care services [48]. Exploring the reason for the ethnic difference in services utilisation authors conclude that cultural and socioeconomic factors are largely responsible of such a difference. The results imply that a care policy may improve the accessibility of mental health services for immigrant women. They also suggest to employ more ethnic and bilingual care providers. Cultural barriers in detecting problem behaviour in children was explored by a study of Crijnen and colleagues [49]. Turkish immigrant teachers reported high levels of anxiety and depression in immigrant Turkish children which go largely undetected by their Dutch teachers.

### - Italy

• A community survey using the standardised clinical interview Present State Examination [24,50] demonstrated that Senegaleses travelling salesmen living in Sardinia, whose working conditions facilitate a community lifestyle, do not appear to be at risk for depression when compared to Sardinian controls. Unexpectedly, higher rates of anxiety and depressive disorders were shown in the few fellow-countrymen who had managed to obtain a steady job with regular wages. In the latter case, the onset of psychopathological disorders was closely associated with the loss of contact with fellow-countrymen. A sample of Moroccan emigrants employed in similar occupations was characterised by a higher risk respect to a population of Senegalese salesmen, and an increased incidence of psychopathological episodes in the six months following their arrival in Sardinia. The authors argued how elements of cultural cohesion such as those represented by the associations of Islamic confraternities may exert strong protective factors against the development of psychopathological, particularly depressive, disorders in immigrants from Senegal.

• A study evaluated the frequency of ICD-10 psychiatric disorders in a community sample of subjects of Sardinian origin resident in Paris. Results were compared to data obtained from a sample of the general Parisian population and from a sample of Sardinians resident in Sardinia [51]. Migration was shown to be associated with a higher risk both of anxiety (as people living in Sardinia) and depressive disorders in the young people (as Parisians). The young emigrants and the children of emigrants (2^nd ^generation emigrants) seem to be prone to drug-abuse and bulimia. The presence of a confidential relationship appears to have a protective effect as shown by the much higher incidence of depression in emigrants who are widowed, separated or live alone. This suggests the need for support strategies.

• Elderly Sardinian residents who had experienced migration are characterised by an increased risk of dysthymia [52]. The problems of adjustment of the return migrant, particularly in the elderly is presented in some Italian papers [53,54]. The focus is on immigration from southern Europe and Turkey toward northern European countries and on progressive aging of people migrated in the 50s and 60s. Authors affirmed that little is known about the health of migrants once they return to their country of origin but the issue represents a very relevant health problem. A recent community survey found a higher frequency of depressive disorders in the Sardinian immigrants in Argentina [55]. Female (not male) showed a higher risk in respect to Sardinians resident in Sardinia. Results seem to suggest that previous emigration in a country that decreased dramatically their economical level may predispose subjects for depressivedisorders comparing with their native population and with subjects migrated in countries more economically advantaged because the lifetime rate of depressive episodes in the Sardinian immigrants in Paris, as reported in a previous above cited research, was lower than in Sardinian immigrants in Argentina. The study suggests the need of systematic researchers and support for European citizen migrated to south America and other economically disadvantaged countries.

#### - Germany

• Hansen and coll. [56] attempt to evaluate if the elevated rate of schizophrenia among migrants has been explained in part by possible misdiagnosis measuring the extend of misdiagnosis among Turkish immigrant patients and German controls. Three investigators (a researcher of Turkish origin, a German researcher and a clinician) evaluated independently two sample of patients one of Turkish an one of German origin. The rate of potential misdiagnosis was higher among migrant, yet not strongly correlated to poor secondary language proficiency. The same research group [57] found in a Turkish of schizophrenic patients a higher rate of depression and hostile excitement than in German schizophrenic patients. Authors say that such a figure may be mainly due to diagnostic differences.

• To solve the problems of language and culture barriers that raise problems for the diagnostic and therapeutic process, Grube developed a bilingual setting by integrating a Turkish psychologist who belongs to a counselling centre into a therapeutic team [58]. Comparing outcome measures in Turkish patient against non Turkish migrants as controls, results showed shorter periods of hospital treatment for Turkish non-schizophrenic in-patients and Turkish schizophrenic patients achieve higher level of rehabilitation. Turkish patients with all kinds of psychiatric diagnoses showed lower ratios of readmission.

• A study on psychiatric inpatient in Frankfurt a.M. found suicidal attempts more frequent among the Mediterranean girls than among their German counterparts [59].

### - Sweden and Scandinavian countries

• The issue about increasing risk for schizophrenia and severe mental disorders in immigrants was studied by several Nordic authors. Hjern and coll [60] found in one of the first longitudinal studies analysing incidence rates of first hospital admission, that five and second generation immigrants have an increased risk for severe psychiatric disorders compared to natives. The highest risks were found in Finnish first-generation immigrants. First generation immigrants from refugee countries have lower risk for alcohol or substance abuse than natives. However the risk in second second-generation refugees is significant higher than in natives. First generation immigrants from refugee countries have the same risk for schizophrenia as native while the risk in second generation refugees is higher than in natives. A complex model integrating both the "Goalstriving stress hypothesis" (the level of psychological stress which derived from a discordance between the goals to be reached and the degree of aspiration) [61] and the cited hypothesis of Eaton and Harrison (concerning executive function abilities and developmental task for formulating the life plan challenges which may be weaker in individuals vulnerable to schizophrenia and which may be made more difficult by the position of disadvantaged ethnic minorities, raising the risk of schizophrenia) [34] may be taken in account. The role of ethnic factors in the risk of schizophrenia was underlined in a study carried out in Malmo [62]. This work confirmed that compared with those who were native-born, migrants had increased risk for Schizophrenia like psychosis. But the risk was most markedly increased in immigrants from East Africa. On the contrary background factors as extreme duress of migration did not appear contribute strongly to increasing risk.

• Also data from Danish Civil Registration System seem to confirm the association between migration and schizophrenia both in immigrants to Denmark and among Danish with a history of foreign residence.

• A paper explored structures of illness meaning among somatizing-Turkish-born migrant women living an a poor and low status suburb of Stockholm [63]. Migrants communicated distress by concrete expression about the body, emotion, social and life situation. Pain was prominent and often lateralised to one side of the body. The use of traditional expression of distress ranged from open use of avoidance. Attribution wascharacterised by verbalising links of coherence between health and aspect of life. Psychiatric attribution was rarely accepted or valued as tool for recovery, or as helpful in linking bodily symptoms to emotional distress. The results of this study point out the mutual need of exploring meaning in the clinical encountered to help patients, particularly migrant, make sense out of different perspectives of illness and healing.

• Compared with Swedish immigrant from Iran, Chile, Turkey, Poland Kurdistan (but for this group differences being not significant) had an increased risk of self reported longstanding psychiatric illness and for intake of psychotropic drugs. Living alone, poor knowledge of the Swedish language, non employment, and low sense of coherence were strong risk factor for self reported longstanding psychiatric illness and for intake psychotropic drugs [64]. Country of birth was a significant risk for poor self reported health and psychosomatic complaints in women of reproductive age in Sweden. Swedish born (but not Finnish) women and female refugees reported more psychosomatic complaints in the 90s than in 80s [65]. Similarly to the cited study on Sardinian immigrants to Paris, the results do not appear to confirm the clinical findings of "somatization" as a privileged "psychopathological course" in latin immigrants reported in the past [66].

• The alcohol related disorders in immigrants was studied by a register based work on a national cohort of 1.25 million youth born 68–79 and 1.5 million of adults born 1929–65 [67]. First and second generation immigrants from Finland (but not from Southern and middle East Europe and non European countries) had a higher relative risk for hospital admission because of an alcohol related disorder compared to the Swedish population. Authors say that patterns of alcohol abuse in the country of origin are strong determinants of alcohol related disorders in first generation immigrants. The patterns in second generation immigrants are influenced by parental countries of origin as well as pattern in the majority of population. The Finnish minority and intercountry adoptees are of particular concern in prevention. Intecountry adoptees have also a high risk for severe mental health problems, suicide and suicide attempts [68].

• A registry study in the county of Copenhagen indicated an excess of Mental Retardation in children from ethnic minorities. The cause are not known and not are aetiological factors for Mental Retardation for a great part of the children. Consanguinity is likely to be a risk factor for Mental Retardation [69].

### - Spain

• Excluding the data from obstetrics and paediatrics, depressive disorders were the second cause of medical consultations in "undocumented" immigrants in the district of Usera-Villaverde, Madrid [70]. In a random sample of immigrant psychiatric patients, depression was more frequent than in the control group of native psychiatric patients, and alcohol abuse less frequent [71]. Among the clinical studies carried out in Spain, there is the annual report of the SAPPIR (Psycho-patological and Psychosocial assistance service for immigrants). The centre focuses its research by symptoms instead of diagnosis. This is due to the fact that in most cultures of origin of immigrants who visit the service, emotion is expressed through the body, but without alexithymia. This approach, which integrates mind and body, favours that depressive symptomatology come along with/is accompanied by somatic symptomatology. According to SAPPIR's research findings, the most frequent symptomatology in immigrants is the triad insomnia – fatigue – migraine [72].

### - Belgium

• Findings of a study carried out in Brussels [73] conformed increased incidence of psychosis in second generation Moroccan immigrants to Belgium.

### - France

• French papers on migration studied more frequently descriptive psychopathology about interpretative psychology than epidemiology. Several authors who had studied the emigration of Islamic populations in France, indicated how many individuals were affected by a certain degree of conflict respect to their migrant situation, with particular concern for the dispositions laid down by the Koran which prohibit leaving the country of origin for non-religious reasons [74]. Problems and conflicts concerning the failure of integration of Moslem French immigrants, particularly Algerians who chose French nationality in 1962, has been taken up in 50 case histories reported by Pouget and coll. [75].

• More recently two cross-sectional studies on perceived health were performed in the Comoro Island and in South Eastern of Marseille. Immigrants in Marseille have higher perceived health status than those living in Comoro Islands for the dimension of physical, mental and general health. The perceived health score level of migrated people is closed to those reported in France [76].

### - Ireland

• Ireland became only recently a destination for migration, thus studies about immigration are still rare. One of the last report about emigration for Ireland, studying attitudes toward emigration found men who contemplated emigration reporting higher self-esteem scores, and women contemplating emigration reporting lower self esteem scores. Women who contemplated emigration had higher depression scores than women who did not contemplate emigration. This pattern was not evident for men. The results indicate that psychologically women view the prospect of emigration less positively than men [77].

• These different attitudes toward emigration in men and women suggest a heuristic hypothesis explaining the different risk in migration (gender determined) shown in the above mentioned Sardinian studies. That hypothesis can be taken into account if similar attitudes will be demonstrated in another catholic but Mediterranean culture. The study on Sardinian immigrants in Argentina suggests that emigration to a country which subsequently suffers dramatic economic problems may increase risk for depressive disorders (but only in women) which are not found in emigrants to economically stable countries. In the survey on Sardinian immigrants in Paris, only the lifetime rate of Depressive Episode in young men immigrants in Paris was higher than the lifetime rate of Depressive Episode in young men Sardinian resident in Sardinia.

• It is very important to underline the two different diametrically opposite economic conditions in which the two migratory waves occurred between Paris and Argentina. Paris offered successful work opportunities but also a new world based on competition. Even though France is closer to Sardinia than Argentina there was a risk, specially for young people, to lose their traditions and family ties [78].

• The hypothesis that arises from the data analysis of the Sardinian and Irish study is that migrated women, with a possible more frequent "depressive" coherent interpretation of self and knowledge [79] may be higher involved during difficult situations when the family safety is under the threat of economical instability. Men may be more at risk in situations of rapid improvement where the competitive challenge becomes pressing as the risk of "goal striving stress" [61].

• The hypothesis of men sensitivity to goal striving stress may be applied to understand the way Ireland with best European economic performance during the period 1980–2000 (the only European country with both increased mean income and decreased unemployment rate) present a suicide male increase around 130%. During the last twenty years period suicides decreased in all Europe but not in the Irish men, instead, the Irish female suicide rate decreased.

• Consistent with constructivist cognitive concepts [79,80], a "depressive style of knowledge" would imply a system whereby the individual explicitly attempts to maintain a coherent image through application of a theory which contemplates a pessimistic view of the world and the future, assuming a sort of "defeat-oriented hyper-responsibility", tacitly challenging the losses experienced.

• Paradoxically depressive attitudes, in face to stress conditions of social change may exert a protective factor if the "compulsive self-responsibilisation" towards the newly-originated socially successful opportunities may modulate the search of new roles which are able to provide subjects with an adequate income and with a sense of leadership whilst at the same time to maintain a role which is more socially accepted and which is perceived as being more "traditional". Several interesting lines of psychosocial research have in fact hypothesised that the cultural transmission tends to be perpetuated when the particular cultural institutions (in this case the social role) are perceived by the individual (and by the other members of the group) as an integral part of the evolving self, but which at the same time, are able to meet new needs and requirements [81]. The latter hypothesis was elicitated to explain the absence of conflicts and the lower risk for depression in a sample of women employed as nurses (a innovative role but that preserve traditional roles) in an African society in rapid change [82].

### - Portugal

• Literature on immigration in Portugal is at present inconsistent. Most international papers are still interested in immigration from Portugal. The study of Ferron and Coll. in Switzerland [83] found in labour migrant adolescents (from Portugal, Spain, Italy, Turkey and former Yugoslavia) lower health behaviour (notably sexual behaviour and substance abuse) than Swiss counterpart. Only alcohol consumption and drink driving place migrant adolescent in a lower risk than their Swiss peer.

• An example of an interdisciplinary approach to provide a framework for understanding and promote mental health in the context of cultural diversity in a Canadian perspective for Portuguese immigrant is presented in a paper from James and Prilleltensky [84]. The framework is not only limited to assessing the needs of individual but draws on anthropology, philosophy, political science, and religious studies to understand the social, cultural, moral, and religious domains. The need for observation in the specific group ethnic model predicting depression in immigrant women (with specific attention to Portuguese) in an a flexible, individualized approach to ethnic women's psychology health care is suggested by Franks and Faux in their Ontario Study [85].

### - Greece

• More than ten years ago a series of very relevant papers appears in the international literature about immigration from Greece. Literature on immigration in Greek is at present inconsistent. The work from Mavreas VR and Bebbington PE [86] shows that the rates of psychiatric disorders in two Greek sample (one of which of Greek Cipriots living in Camberwell, London and the other one living in Athens) were somewhat higher than those of the Camberwell population. Greeks reported more symptoms of general anxiety disorders. This is consistent with the results others researches and with the Sardinian immigration studies in which a higher frequency of anxiety disorders in the Sardinian sample and of depressive disorders among Parisians was observed. Mavreas and Bebbington suggest a greater risk of anxiety disorders in southern and of depression in northern European countries.

• A paper from Fitcher and coll. [87] found significantly higher GHQ-28 scores for Greeks and Turks adolescents in their homeland as compared to Greek adolescent in Munich. Results seems do not confirm the acculturation stress hypothesis.

### - Austria

• A paper describes the behaviour disturbance and emotional problems of Turkish immigrant and Austria children aged 9 to 13 years living in Vienna, rated by their parents and both by Turkish and German teachers. The prevalence of behaviour problems did not differ between the Turkish and Austrian children [88].

• A paper presented at the DTGPP Satellite Conference, Ethnicity & Mental Health in Europe, Essen Germany, September 2003 [89] deals with the issue of the misdiagnosis of schizophrenia in migrants, authors think that hebephrenic schizophrenia in migrants may besometimes misdiagnosed as chronic depressive status due to cultural barriers.

### - Drug abuse

• Reports to investigate the reasons for drug abuse among immigrant youth have been carried out in Sweden [90], France [91-94] and Germany [95] coming up with similar conclusions which suggest that drug abuse was a consequence of difficult social integration. A 1996 WHO report noted that the consumption of tranquillisers and antidepressants by young immigrants across Europe is growing. Alcohol abuse is also reported as an important problem among immigrants population in Europe.

• A recent review of the literature underlined that the association between migration and addiction is very heterogeneous. More or less drug and alcohol dependence than native population have been reported in different migration phenomena across the world [96] As suggested in some above cited studies on alcohol abuse, but probably not with the same strong association, patterns of addiction abuse in the country of origin are determinants of alcohol related disorders in first generation immigrants. The author of the cited lecture indicates, in spite of the chief role of migration drug problems in public debate and concern, a lack data about alcohol dependence in the migration population in Europe. The report of the European Union Strategy on Drugs 2000–2004 has no mentions or suggestions of these specific problems in any European Union country.

### - Refugees and mental health

• Recent surveys have shown that two thirds of refugees experience anxiety and/or depression [97].

• Refugees have a high incidence of post traumatic stress disorder, depression, anxiety, panic disorder and agoraphobia [98].

• Shortages of food, being lost in war situation, being close to death and suffering serious injury were each related to specific psychiatric symptoms in a community sample of adult Somali refugees [14].

• A relevant work on psychiatric disorders on Bosnian refugees was performed by the Harvard USA program in Refugee Trauma [99,100] The initial study reported a high rate of disabling depression and post traumatic stress disorder among refugees. Bosnian Refugees followed up initially found asylum in Croatia in 1996 and now returning in Bosnia or remained in the Balkans (70.4%9) or migrated in European Union or USA (21.3%), the others had died (21.3%) or were lost to follow-up [100]. Nearly 50% former Bosnian refugees who remained living in the Balkan area still present psychiatric symptoms and disability 3 years after initial assessment. Depression was unremitting, disabling, and potentially associated with premature death in the elderly. About 20% of those did not have symptoms of psychiatric disorder at starting time had symptoms at follow-up. Depressed refugees had three times the risk of dying than non depressed. Refugees who emigrated were more traumatised, better educated and had fewer health and mental health problems than the majority who remained in the region.

• A recent lecture [46] on global risk and protective factors in the development of mental disorders in refugees cutting across lines of social class and cultural identity, analysing a community based study on Iraqui refugees in The Netherlands, suggests that a long asylum procedure is associated with psychiatric disorders. And indicates that both policy makers and mental health workers should take note of this findings.

#### ESEMeD

The recent Study of the Epidemiology of Mental Disorders – **ESEMeD**, a survey carried out in six European countries (France, Belgium, Italy, Spain, The Netherlands, Germany), could mean an important contribution for the study of epidemiology of mental disorders in immigrants living in Europe. The ESEMeD's sample consisted in a random selection of 21.425 individuals from the general population aged 18 or older and non-institutionalised. This sample comprises a 6.03% (6.58%weighted) of foreign individuals. The study will provide us with highly relevant information concerning prevalence and comorbidity of mental disorders, and patterns of service utilization of these populations.

The recent Report "The State of Mental Health in Europe" [101] compared the ESEMED findings for psychological distress, as measured by the SF-12 questionnaire, for people who were born in the country compared to those who not born in the country. Because of the way the sample were designed, it was not possible to compare those born in the country with those not born in the country for Italy. The country ratios (risk of distress in immigrants against natives) were: 1.0 in Belgium, 1.2 in The Netherlands, 1.4 in Spain, 4.3 in Germany, 4.7 in France.

## 4. Health policy toward immigrants: access to health care services

Despite in the migrants groups vulnerable populations were identified with respect to health problems, in many European countries there are migrants who fall outside the existing health and social services, something which is particularly true for asylum seekers and undocumented immigrants (due to the fact that most asylum applications are refused, the division line between both situations is very small). They are usually only entitled to emergency health services.

For example, exclusionary policies in relationship of access to health services on behalf of asylum seekers have been documented in France, where limitations in access were found for those asylum seekers who are not granted a work permit, even if they can't work due to ill health or language barriers [102].

In spite of some states efforts to universalise the right to access national health care services, therefore including undocumented migrants there exist an important lack between rights and accessibility [20]. Undocumented immigrants may not access health care services for causes such as administrative obstacles or fear of being reported to the police. This action is not in practice nowadays in most countries, but nevertheless certain evidence of it exists. According to the study Easy Scapegoats: Sans Papier immigrants in Europe [103], the German Law of Foreigners obligates public institutions to denounce undocumented people to the Office of Foreigner's Affairs. Some hospitals have informed the police while treating undocumented immigrants.

Other obstacles immigrants in general may find when attempting to seek for health care in European countries despite their legal situation are the lack of adequate information about the available health care facilities and communication (language) problems. Moreover, in the case of psychological problems, it may result an obstacle the fact that mental health problems may lead to stigmatisation of that group.

Nevertheless, the health care gaps that are being left by the authorities are being covered by the informal work of doctors at the health system and by NGO's, which provide with medical and, specially, mental health assistance together with health promotion and prevention programs among other services.

## 5. State of the art in Mental Health Care provision

Researches in the field of access to care deal with subjective and objective barriers. The objective barriers either lie in the domain of the available information, the structure of the health care system, as well as the availability of treatment modalities. The subjective barriers lie either on the side of affected person, the treatment professionals orhealth care planners. Ethnic minority groups, particularly migrants, are faced with several potential barriers in the access of care, resulting in a lower representation in mental health services [56].

Taking the above into account, an examination of studies of mental health services for migrants groups in Europe shows that quality and availability of this provision is very patchy between countries. For example, a mapping exercise of mental health care in Europe carried out by Watters [104] suggested a serious absence of mental health care for migrants in Central and Eastern Europe, something of great importance if we take into account the rapid economic and social changes affecting these regions [104].

Delivering of specific mental health care services for migrants is integrated into mainstream mental health services, who provide the majority of these, in some countries (Netherlands, United Kingdom) even though there exist parallelly specific community services mainly funded by the government. In other countries, these specific community services and NGOs are the main providers of mental health services for these groups while the national health system adapts to the new health needs in a very slow manner (Spain, Portugal). Nevertheless, even those countries with a longer experience of migration and where the bulk of mental health services are being provided by the statutory sector, denounce the weakness of this provision. Most important weaknesses are reported next.

Communication problems are especially significant as a factor that generates exclusion and have been reported in all countries studied. Language is the most essential work instrument of the mental health care provider as the analytical and therapeutic processes are heavily dependent on language as a medium. Drawing on recent studies, services are almost always only available in the majority language [104,7]. The Health for all, all in health study [104] reported that most migrants surveyed tended to think that care providers underestimate the language problems and also say that language difficulties make them more aggressive and paranoid towards the care provider.

The diagnostic process is complicated by the cultural differences between the immigrant and the therapist. Diagnostic mistakes are more often than with native patients. It is important to consider the immigrant's ethnic identity as well as his lay models explaining his illness. Furthermore, his expectations about the treatment must be considered if the treatment shall be successful. Integration of immigrants into a developed network of psychiatric care system may be a useful strategy. Another condition for a more effective psychiatric care of immigrants is a well foundedtranscultural psychiatric training of the professionals [105].

There is a clear deficiency in training and education practices on the subject of understanding immigrant culture for mental health professionals working with migrants, as every country makes the demand of this special training. If well it is true that some countries of Northern Europe have a major and longer experience in this realm, Fernando [106] stresses the necessity of urgent change in the training of professionals, the way mental health assessments are made and the narrow eurocentric nature of therapy. It has been reported that, in many cases, psychosocial problems can mislead the health care staff who are not familiar with the process and impact of migration on psychological health [107].

On the other hand, there exists a big demand for the assistance of interpreters or health agents – who are under utilised – so that migrants are not force to rely on friends and family members to act as interpreters, a practice which has been described by the head of one transcultural psychiatric unit as unethical and totally unacceptable [104]. However, immigrants often feel discomforted because they suspect the interpreters to disclose private and intimate information to others. Some health problems are difficult to be discussed in presence of an interpreter or a member of the family [108]. Therefore, research into working with interprets or health agents and studies of the processes of transmission and counter-transmission are also required.

Shortages in the provision of appropriate training of professional groups could be addressed through the involvement of service users from migrant groups, who could help to overcome the problems of racial and cultural stereotyping which have also been reported in many services.

The absence of monitoring and consultation with clients, which makes impossible for planners and providers to evaluate the suitability of services, are other deficiencies that characterises mental health provision for migrants in Europe.

In order to address all these deficiencies, it is necessary to provide with an adequate financing and a continuity of the grants for research into the multicultural health demand. Most of the projects accomplished have been characterised for a short-term funding.

Finally, there is to highlight the importance of adopting an integrated approach to mental health care that moves away from psychiatric care only, as it has been stressed in a recent report of the WHO in collaboration with Red Cross and Red Crescent organisations.

## Competing interests

The author(s) declare that they have no competing interests.

## Authors' contributions

MGC, MB, MCH and JMHA conceived of the manuscript, and drafted it. All authors read and approved the final manuscript. The "Report on the Mental Health in Europe" working group discussed the manuscript during a meeting.
